# Exosomes derived from chemically induced human hepatic progenitors inhibit oxidative stress induced cell death

**DOI:** 10.1002/bit.27447

**Published:** 2020-06-30

**Authors:** Sujin Hyung, Jaemin Jeong, Kyusoon Shin, Ju Young Kim, Ji‐Hye Yim, Chan Jong Yu, Hyun Suk Jung, Kyung‐Gyun Hwang, Dongho Choi, Jong Wook Hong

**Affiliations:** ^1^ Center for Exosome & Bioparticulate Research Hanyang University Gyeonggi‐do Korea; ^2^ HY Indang Center of Regenerative Medicine and Stem Cell Research Hanyang University Seoul Korea; ^3^ Department of Surgery Hanyang University College of Medicine Seoul Korea; ^4^ Department of Bionanotechnology, Graduate School Hanyang University Seoul Korea; ^5^ Division of Chemistry and Biochemistry Kangwon National University Chuncheon Korea; ^6^ Department of Dentistry/Oral & Maxillofacial Surgery, Collage of Medicine Hanyang University Seoul Korea; ^7^ Department of Bionanoengineering Hanyang University Gyeonggi‐do Korea; ^8^ Department of Medical & Digital Engineering Hanyang University Seoul Korea

**Keywords:** antioxidant activity, cell survival, exosome therapy, human hepatic progenitor cells‐derived exosome (EXOhCdHs), liver disease

## Abstract

The emerging field of regenerative medicine has revealed that the exosome contributes to many aspects of development and disease through intercellular communication between donor and recipient cells. However, the biological functions of exosomes secreted from cells have remained largely unexplored. Here, we report that the human hepatic progenitor cells (CdHs)‐derived exosome (EXO^hCdHs^) plays a crucial role in maintaining cell viability. The inhibition of exosome secretion treatment with GW4869 results in the acceleration of reactive oxygen species (ROS) production, thereby causing a decrease of cell viability. This event provokes inhibition of caspase dependent cell death signaling, leading to a ROS‐dependent cell damage response and thus induces promotion of antioxidant gene expression or repair of cell death of hypoxia‐exposed cells. Together, these findings show the effect of exosomes in regeneration of liver cells, and offer valuable new insights into liver regeneration.

## Graphical abstract

Protective effect of hCdHs‐derived exosome against oxidative stress in hepatocyte. Exosomes were isolated from chemically induced human hepatic progenitors (hCdHs) by using ‘H’ method. hCdHs‐derived exosome (EXOhCdHs) has a beneficial effect on antioxidant activity of damaged hepatocytes.

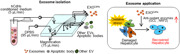

## 1 INTRODUCTION

The liver, among all the body organs, possesses a unique regenerative capacity to repair its structure, size, and functions in response to the loss of hepatic tissue caused by partial surgical removal, toxins, or infection. In the condition of hepatocyte pathologic change, the majority of cells in the residual liver rapidly re‐enter the cell cycle accompanied by the induced expression of a variety of growth‐promoting genes. This prominent regeneration capacity leads to a foundation of potentially clinical consequence for patients after hepatic injury, cancer reaction, infectious, or toxic injury (Abu Rmilah et al., 2019; Kholodenko & Yarygin, 2017; Raven & Forbes, 2018; Taub, 2004). Although this intrinsic ability for regeneration can restore the damaged liver, complete functional recovery of the injured liver organ is required as a compensatory response managing the disturbance of homeostasis of the whole body induced by functional deficiency. Furthermore, defective liver regeneration contributes to the pathogenesis of liver cirrhosis, primary liver cancer, and liver failure (Bae, 2008; Fox et al., 2014; Sturesson, Nilsson, Eriksson, Spelt, & Andersson, 2013; S. J. Yu, 2016). To achieve functional restoration and minimize the risk of postoperative liver failure, the patient status and regenerative conditions after liver damage must be fully considered. Recent studies have shown that prominent methods including cell source (e.g., injection or transfusion of cell suspensions, Banas et al., 2007; Basma et al., 2009; Cameron et al., 2015; Chen et al., 2012; Hong et al., 2005; Touboul et al., 2016; J. Yu et al., 2007; humoral factors, e.g., growth factors, cytokines, Bohm, Kohler, Speicher, & Werner, 2010; Galun & Axelrod, 2002; Ibrahim & Weiss, 2019; Michalopoulos & Khan, 2005), and extracellular matrix‐based technology (e.g., decellularization, De Kock et al., 2011; Klaas et al., 2016; Mazza et al., 2015; Pla‐Palacin, Sainz‐Arnal, Morini, Almeida, & Baptista, 2018) are remarkable regeneration resources; however, little is known regarding the molecular mechanism underlying spontaneous liver regeneration. More advanced strategies are required to better understand the molecular mechanism of regenerative therapy.

Exosomes, endosomal membrane vesicles ranging in size from 30 to 200 nm, have emerged as key regulators and promoters of intercellular communication between donor and recipient cells through transfer of information via their cargo, which involves nucleic acids, proteins, lipids, messenger RNAs, and microRNAs (Aharon, Rebibo‐Sabbah, Tzoran, & Levin, 2014; Boyiadzis & Whiteside, 2017; Levy, 2017; Simons & Raposo, 2009; Thery, 2011). Exosomes originate from the late endosomal compartment of donor cells from the inward budding of endosomal membranes and deliver genetic cargo to the target cell (Simons & Raposo, 2009; Thery, 2011). Recently, emerging evidence has supported the fact that exosomes play essential roles in intercellular communication between cells, serving as a vehicle for transferring a variety of cellular constituents including protein and RNAs (Hessvik & Llorente, 2018; Simons & Raposo, 2009; Thery, 2011; Zhang, Liu, Liu, & Tang, 2019). Stem cell‐based exosomes can influence tumor development, liver functions, and the viability of neighboring cells, and are involved in the regulation of proliferation and apoptosis of hepatocytes, and alleviation of oxidative stress (Basu & Ludlow, 2016; Deregibus et al., 2007; Phan et al., 2018; Royo & Falcon‐Perez, 2012; Tan et al., 2013; Yanez‐Mo et al., 2015). Despite the significant advances in liver regeneration, the biological activity of these cells‐derived exosomes seems to be impeded by dependence on donor‐cell conditions including rapid loss of differentiation potency, low efficiency of hepatic differentiation, and low rate of proliferation in culture. Exosomes that derived from the advanced cell with regenerative potential are required for enabling functional regeneration and repair of diseased livers.

Recently, we showed that bipotential human hepatic progenitor cells (hCdHs) reprogrammed from human primary hepatocyte treatment with a mixture of additional components including A83‐01, CHIR99021, and HGF have a potential capability in promotion of both proliferation and differentiation (Kim et al., 2019). However, the precise function whether hCdHs is essential for regeneration of damaged liver, and how hCdHs elicited cell protection through secreted soluble proteins or vesicles, remains unclear. In this study, we found that the hCdHs‐derived exosome (EXO^hCdHs^) has a beneficial effect on antioxidant activity of damaged hepatocytes. Moreover, we provide evidence that the EXO^hCdHs^ contribute the expression of cell death‐related to proteins, indicating that EXO^hCdHs^ contains components correlated to cell protection. Together, our results suggest that EXO^hCdHs^ contribute to liver regeneration by supporting the survival of damaged liver cells after injury.

## 2 MATERIALS AND METHODS

All procedures pertaining to the acquisition of biological samples were approved by the Institutional Review Board of Hanyang University (HYI‐16‐229‐3), and all experiments were conducted in accordance with the guidelines and regulations stipulated by the committee. Human liver tissues were obtained from donors operated on in Hanyang University Medical Center.

### 2.1 Cultures of human chemically derived hepatic stem cells (CdHs) and immortalized hepatocyte

Human primary hepatocytes for CdHs generation were isolated using a two‐step collagenase perfusion method. Hepatocytes (1 × 10^5^ cells) were seeded on collagen‐coated dishes, and cultured with 4 µM A83‐01 (Gibco), 3 µM CHIR99021 (Stemcell Technologies), and 20 ng/ml of HGF (Peprotech) for 14 days as described before. Hepatic stem cell markers were evaluated by immunocytochemistry and quantitative reverse‐transcription polymerase chain reaction (qRT‐PCR).

The Fa2N‐4 cells (Sekisui XenoTech, KS) were derived from a 12‐year‐old Caucasian female donor with the SV40 large T antigen. Fa2N‐4 hepatocytes were cultured with basic high glucose DMEM/F‐12 Media (Gibco, CA) containing 10% FBS (Gibco), 10 mM nicotinamide (Sigma‐Aldrich, MO), 0.1 μM dexamethasone (Sigma‐Aldrich), 1% insulin‐transferrin‐selenium (Gibco), 1% penicillin/streptomycin (Gibco), and 20 ng/ml epidermal growth factor (Peprotech, NJ). The cells were maintained at 37°C with 95% humidity and 5% CO_2_. Fa2N‐4 cells were plated in collagen‐coated well plates or dishes.

### 2.2 Exosome depletion assays as a control

To assess the depletion of exosomes, cultured hCdHs were treated with 10 µM of GW4869 and hCdHs‐conditioned medium was harvested after 48 hr. hCdHs‐conditioned medium with 10 µM GW4869 was separated using a “H” method and exosome protein levels were analyzed.

### 2.3 Exosome purification

We isolated exosomes from the conditioned medium of hCdHs (Kim et al., 2019) using an innovative method called the “H” method (Shin et al., 2017). The “H” method not only separates nanometer‐sized exosomes from different sized particles in cell conditioned medium or various body fluids without damage, but also enables the selective separation and concentration of exosomes and nanoparticles through precise control of the flow rates of several solutions. The “H” method is a breakthrough that separates nanometer‐scale particles of different sizes by exploiting the laminar flow phenomenon observed when a sample passes through a micrometer‐scale space, and the unique physical behavior that depends on particle size. With this method, 30–200 nm exosomes and 100–1,000 nm apoptotic bodies can be collected through different outlets (Shin et al., 2017). The microfluidic system consists of two inlet channels for the sample and buffer, nine outlet channels, and one highly specific magnification channel that continuously separates undamaged exosomes through precise flow rate adjustment. The flow rates of the sample inlet channel, buffer inlet channel, and magnification channel are in a ratio of 5:95:75 respectively. In this experiment, a conditioned medium of hCdHs was injected into the sample inlet channel, while the buffer was injected into the buffer inlet channel. Depending on the flow rates of the injected conditioned medium fluid and buffer, a pinched flow of a specific width is formed. At this point, we can create a limited flow region that is formed by finely controlling all particles around the wall of the microfluidic chip and around the pinched flow. In this limited flow region, the particles in the sample gather. Subsequently, particles present in the ordered sample migrate to the broadened channel region, and are reordered by size as they pass through the area. At this time, separation in the strict sense has already occurred. When the particles in this confined sample pass through a broader channel region, we manipulated the negative pressure through the magnification channel so that it acts at right angles to the lower side of the sample flow. Through this unique precision fluid operation, called “magnification,” the aligned particles follow different paths according to their size, and the physical distances between them in the different paths are further amplified. Through this very precise process, we can obtain nanometer‐sized bioparticles by selective separation from outlets at different physical locations depending on their size. When this process was operated under the same conditions as previously, 30~200‐nm particles, that is, the size of the exosomes described in Figure S1, gathered at outlet channels 1–3 (Shin et al., 2017), while larger microvesicles and apoptotic bodies gathered at outlet channels 4–9. The flow rates of the injected sample and buffer solution were 5 and 95 μl/min, respectively, and that of the buffer removed by magnification was 75 μl/min. As shown in Figure S2, under these conditions, processed samples were obtained through the nine outlets at a total rate of 25 μl/min. Besides this, under the conditions in Table S1 and Equation S1, 500 μl of separated solution containing pure exosomes with no physical or chemical damage was collected over 1 hour.hr from 300 μl of culture medium that contained cell debris, apoptotic bodies, microvesicles, and exosomes. The system is kept constant at 4°C using a cooling system to prevent degeneration of the exosomes during the separation process, including sample preparation, before, during, and after separation. In this study, hCdHs were cultured for about 2 days using a special culture medium (Kim et al., 2019) in a culture dish. The culture medium was harvested to separate undamaged exosomes when cell growth reached about 80%, at the most active growth point.

### 2.4 Nanoparticle tracking analysis (NTA)

To determine the size of the isolated sample, NTA was performed using an LM10 (NanoSight) instrument. Freshly separated vesicles were diluted with filtered PBS and 15–20 vesicles were recorded for each fraction. Each sample was subjected to a red laser (642 nm) for 1 min three biological replicates. Data were analyzed using NTA software (ver. 3.1; NanoSight). All experiments were conducted at room temperature.

### 2.5 Western blot analysis

For western blot analysis, purified exosomes were mixed with 5% sodium dodecyl sulfate (SDS) sample buffer (T&I), and cells were lysed and homogenized in radioimmunoprecipitation assay buffer (T&I) containing protease inhibitors. Protein concentrations were measured using Bradford assays. Lysed samples were separated onto SDS–polyacrylamide gel electrophoresis gels (12% and 15%) and transferred to a polyvinylidene difluoride (PVDF) membrane (Bio‐Rad). Primary antibodies used were cleaved PARP (1:2,000; Cell signaling Technology), Bcl‐XL (1:1,000; Cell signaling Technology), MCL‐1 (1:1,000; Cell signaling Technology), and β‐actin (1:5,000; Santa Cruz Biotechnology), and CD63 (1:500; Novus). The PVDF membrane was blocked with 5% skim milk (BD) in TBST buffer (25 mM Tris, 190 mM NaCl, and 0.05% Tween 20, pH 7.5) for 1 hr at room temperature, followed by treatment with the primary antibody at 4°C overnight. After five 15‐min washes with TBST, the membranes were incubated with the corresponding IgG/horseradish peroxidase (HRP) secondary antibody at a dilution of 1:500–1:2,000 for 2 hr at room temperature, washed, and visualized using the Clarity Western ECL substrate (Bio‐Rad). Band intensities were measured using ImageJ software (NIH).

### 2.6 Transmission electron microscopy (TEM)

Isolated exosomes were fixed with 2% glutaraldehyde overnight at 4°C and then diluted 10‐fold with PBS for electron microscopic observation. The sample was plated onto the glow discharged carbon‐coated grids (Harrick Plasma), which were immediately negatively stained using 1% uranyl acetate solution (Jeong et al., 2018). The samples on grids were analyzed with a Tecnai 10 transmission electron microscope (FEI, Instrumentation was used in the Kangwon Center for Systems Imaging) operated at 100 kV. Images were collected with a 2k × 2k UltraScan CCD camera (Gatan).

### 2.7 Reactive oxygen species (ROS) assay

ROS production was measured using a ROS‐sensitive dye, namely 2′,7′‐dichloro‐dihydrofluorescenin diacetate (H2DCFDA). H2DCFDA is proposed to react with peroxyl products and peroxyl radicals and not with singlet oxygen directly, but singlet oxygen rapidly forms peroxyl radicals and thus can indirectly contribute to the formation of dichloro‐dihydrofluorescein (DCF). For the measurement of endogenous ROS levels in Fa2N‐4, cells were incubated with 10 μM 2′,7′‐dichlorofluorescein diacetate (Molecular Probes, Inc., Eugene, OR) for 30 min, stained with 4′,6‐diamidino‐2‐phenylindole (DAPI). Samples were observed under inverted fluorescence microscopy (IX53; Olympus, Japan). DCF fluorescence intensity was examined in fluorescence images, taken during a single session with the exposure and gain settings kept constant, of samples obtained from one experiment that was processed side‐by‐side. The number of DCF‐positive cells among all cells stained with DAPI was counted in three random fields of view (*n* = 3). Samples were immediately analyzed.

### 2.8 Real‐time PCR

Total RNA was extracted from immortalized hepatocyte cells using a TRIzol kit (Life Technologies) by following the manufacturer's recommendations. The concentration of RNA samples was measured by a Nanodrop ND‐1000 (Therrmo Fisher Scientific). For real‐time analysis, cDNA was transcribed from a total of 100 ng of DNase I‐treated RNA using a SuperScript III Reverse Transcriptase (Therrmo Fisher Scientific) and Oligo DT (Roche Diagnostics, Rotkreuz, Switzerland). Real‐time quantitative PCR amplification reactions were carried out in a Detection system from Bio‐Rad (CA) in a 20 μl volume. To determine relative mRNA expression, the housekeeping gene (GAPDH) and antioxidant marker gene with SYBR green (Dyne Bio) were used. Gene expression was analyzed by performing PCR amplification of cDNA with the specific primers: NRF2 (forward), 5′‐ACA CGG TCC ACA GCT CAT C‐3′ and (reverse), 5′‐TGT CAAA TCA AAT CCA TGT CCT G‐3′; GCL (forward), 5′‐TTG CCT CCT GCT GTG TGA TG‐3′ and (reverse), 5′‐ATC ATT GRG AGT CAA CAG CTG TAT GTC‐3′; GAPDH (forward), 5′‐TGC ACC ACC AAC TGC TTA GC‐3′ and (reverse), 5′‐GGC ATG GAC TGT GGT CAT GAG‐3′.

### 2.9 Immunofluorescence

Cultured hCdHs were fixed at 4 days with 4% paraformaldehyde (PFA; T&I) at 4°C for 20 min and treated with 0.2% Triton X‐100 (Sigma) for 20 min at room temperature, followed by blocking in 4% bovine serum albumin (BSA; Millipore) overnight at 4°C. Fixed samples were incubated overnight with primary antibody diluted in 1% BSA at 4°C, washed three times with 1% BSA and stained with Alexa 488‐ or 594‐conjugated secondary antibodies. The primary antibodies used were anti‐SOX9 (1:100; Abcam) and anti‐CK19 (1:100; Abcam). The secondary antibodies used were goat anti‐rabbit IgG H&L (1:500; Abcam). Nuclei were stained with DAPI (Life Technologies) for 15 min. Samples were observed under confocal microscopy (Ziess LSM810).

### 2.10 Measurement of cell cytotoxicity

To examine cell viability in immortalized hepatocytes, we used the LIVE/DEAD cell staining kit (Abcam) according to the manufacturer's instructions. Briefly, a total of 5 × 10^3^ immortalized hepatocytes were cultured in a well plate and treated with H_2_O_2_ in the presence or absence of EXO^hCdHs^ for 48 hr. Cells were washed three times with PBS, followed by treatment with a mixture of calcein‐AM and propidium iodide in PBS for 30 min in incubator. Samples were washed two times with PBS and examined under a confocal microscope (LSM 800; Zeiss). In each experiment, live and dead cells were counted in five random confocal microscopy images.

### 2.11 Statistical analyses

All statistical analyses were performed using GraphPad Prism software (GraphPad, Inc.). Analysis of variance was performed to compare means among three or more groups, and unpaired two‐tailed *t* tests with Welch's correction were used to compare means between two groups. Data from a minimum of three independent experiments are presented as means ± standard error of the mean (*SEM*). Statistical significance was determined at *p* < .05.

## 3 RESULTS

### 3.1 Separation and identification of hCdHs‐derived exosomes

**Figure 1:**
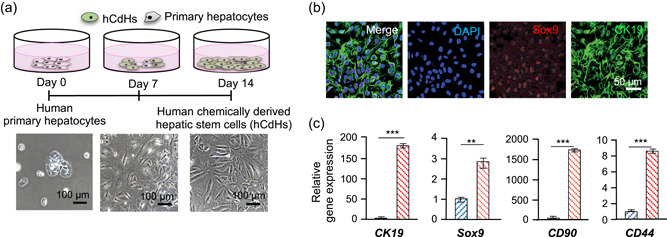
Establishment of the human chemically derived hepatic progenitors (hCdHs) from human hepatocyte. (a) Schematics for hCdHs generation. Human primary hepatocytes were transformed with hepatic stem cell treatment with HGF, A83‐01, and CHIR99021. The morphology of hCDHs became a longitudinal shape from the epithelial morphology of hepatocytes. Scale bars = 100 μm. (b) Hepatic stem cells marker protein expression in hCdHs measured by immunocytochemistry. SOX9 (red) and CK19 (green), which are representative hepatic stem cells markers, are stained and merged in hCdHs. Nuclei were counterstained with Hoechst 33342 (blue). Scale bars = 50 μm. (c) Hepatic stem cells marker gene expression in hCdHs. CK19, SOX9, CD90, and CD44 gene expression determined by quantitative reverse‐transcription polymerase chain reaction (RT‐qPCR). The values are means ± *SEM* from at least three independent experiments, normalized to cell lysate glyceraldehyde 3‐phosphate dehydrogenase (GAPDH) levels. **p < .01, ***p < .001; unpaired, two‐tailed *t* test with Welch's correction [Color figure can be viewed at wileyonlinelibrary.com]

**Figure 2:**
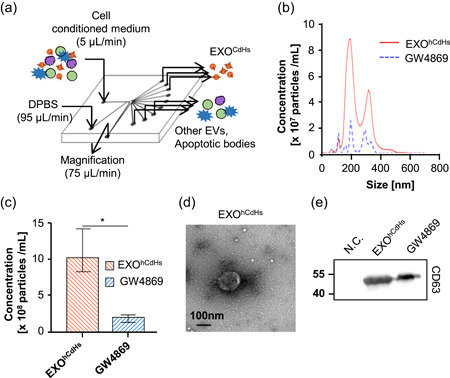
Characterization of human chemically derived hepatic progenitors (hCdHs)‐derived exosomes. At 3 days after hCdHs were changed with exosome‐depleted medium, the conditioned medium was collected from hCdHs and exosomes were isolated from the medium by using the “H” method (a). EXO^hCdHs^ were analyzed and characterized in several experiments by nanoparticle tracking analysis (NTA), western blot (WB) analysis, transmission electron microscopy (TEM). (b) Representative NTA profiles and (c) quantification results of EXO^hCdHs^. The values are means ± *SEM* from three independent experiments. *p < .05; unpaired, two‐tailed *t* test with Welch's correction. (d) Morphology observed by TEM. Scale bar = 100 nm. Representative immunoblot of the exosome surface marker CD63 is shown in (e). hCdHs‐conditioned medium with 10 µM GW4869 was separated using a “H” method. Nanosized vesicles separated from conditioned medium and incubated without cells were used as a negative control [Color figure can be viewed at wileyonlinelibrary.com]

To generate human chemically derived hepatic stem cells, human primary hepatocyte from patient livers were seeded onto collagen‐coated dishes, and cultured with HGF, A83‐01, and CHIR99021 for 14 days. Hepatocytes which cannot proliferate were converted into hepatic stem cells (Figure 1a). As per our previous report, hCdHs expressed hepatic stem cell marker protein, and genes, such as CK19, SOX9, CD90, and CD44, respectively (Figure 1b,c). As shown in Figure 1b, CK19 was expressed in the cell membranes and SOX9 was expressed in the nuclei of the hCdHs. These pieces of data suggest that hCdHs derived from human primary hepatocytes are hepatic stem cells. To isolate the exosomes from human CdHs, the cells were cultured for 48 hr with cultured media containing exosome‐depleted FBS and harvested exosome containing media (Figure S3). EXO^hCdHs^ were isolated from other particles, including microvesicles and apoptotic bodies, using the “H” method, which separated according to the size without big apoptotic bodies and characterized by size and morphology (Figure 2a; Shin et al., 2017). The NTA results showed that the size distribution of EXO^hCdHs^ had a major peak of approximately 100–200 nm although a small number of larger vesicles were included (Figure 2b). As a control, we measured the concentration of isolated exosomes that was pretreated with GW4869 to prevent exosome generation and secretion from cells. Nanosized particles were then separated from hCdHs‐conditioned medium treated with GW4869 using the ‘H'method. Besides this, the number of total exosomes was more than 10 × 10^8^ particles while the concentration of exosome treatment with GW4869 was 1.8 × 10^8^ particles, calculated using an LM10 (Nanosight; Figure 2c). Transmission electron microscopy (TEM) images indicated a similar tendency as the NTA result, showing that hCdH‐derived exosomes had a spherical membrane structure (Figure 2d). Western blot analyses confirmed the presence of comparable amounts of exosomal protein markers CD63 in exosomes isolated from hCdHs (Figure 2e). These results suggest that hCdHs secrete extracellular vesicles that are called exosomes, and only exosome particles without apoptotic particles can be isolated via our own system.

### 3.2 EXO^hCdHs^ increase the antioxidant gene expression and decrease H_2_O_2_‐induced ROS generation

**Figure 3:**
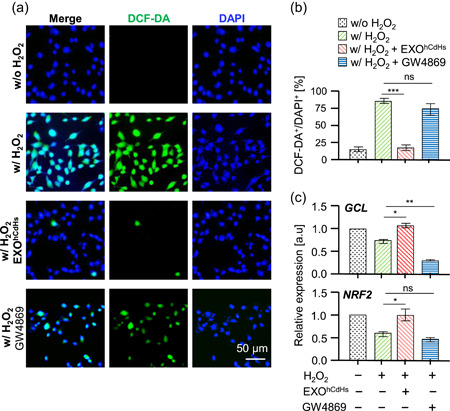
EXO^hCdHs^ regulated intracellular reactive oxygen species (ROS) level of oxidative stress induced hepatocytes. Hepatocytes were treated without or with EXO^hCdHs^ for 24 hr before H_2_O_2_ treatment. The cells were treated with H_2_O_2_ (400 μM) for 6 hr and the concentration of EXO^hCdHs^ was maintained though culturing until analysis. (a) Intracellular ROS levels were determined by dichloro‐dihydrofluorescein diacetate (DCF‐DA) staining under fluorescence microscopy. Scale bar = 50 μm. The quantification result of DCF‐DA‐positive cells per 4′,6‐diamidino‐2‐phenylindole (DAPI)‐positive cells is shown in (b). The values are means ± *SEM* from three independent experiments. ***p < .001 (w/H_2_O_2_ vs. w/H_2_O_2_ + EXO^hCdHs^), ns, nonsignificant (w/H_2_O_2_ vs. w/H_2_O_2_ + GW4869); unpaired, two‐tailed *t* test with Welch's correction. (c) Antioxidant gene expression levels were determined by treatment with H_2_O_2_ for 6 hr with presence or absence of EXO^hCdHs^. Relative mRNA expression levels of indicated genes determined by quantitative reverse‐transcription polymerase chain reaction (RT‐qPCR). The values are means ± *SEM* from three independent experiments. *p < .05 (w/H_2_O_2_ vs. w/H_2_O_2_ + EXO^hCdHs^ of *GCL*), **p < .01 (w/H_2_O_2_ vs. w/H_2_O_2_ + GW4869 of *GCL*), *p < .05 (w/H_2_O_2_ vs. w/H_2_O_2_ + EXO^hCdHs^ of *NRF2*), ns, nonsignificant (w/H_2_O_2_ vs. w/H_2_O_2_ + GW4869 of *NRF2*); unpaired, two‐tailed *t* test with Welch's correction [Color figure can be viewed at wileyonlinelibrary.com]

During a liver transplantation, the interruption of blood flow followed by reperfusion produces an abrupt increase in ROS, disrupting balance and causing oxidative stress and generally inflammatory progression. To mimic oxidative stress in vitro, and evaluate the exosome effect, we treated H_2_O_2_ to SV40 immortalized hepatocytes. With treatment of EXO^hCdHs^ (1:2,000, dilution in cell culture media) in the presence of 400 μM H_2_O_2_, ROS detected with DCF‐DA were markedly decreased (Figure 3a,b) under florescence microscopy. However, the DCF‐DA‐positive cell per all DAPI‐positive cells was enhanced in the GW4869 condition (1:2,000 dilution in cell culture media), and there is no significant difference in the presence and absence of GW4869 in H_2_O_2_ cells. In addition, to investigate whether EXO^hCdHs^ can affect H_2_O_2_ induced ROS generation in hepatocytes, we focused on the transcription factor; nuclear factor erythroid 2‐related factor 2 (NRF2) and its target glutamate cysteine ligase (GCL) involved in regulating ROS production. NRF2 is a potential regulator of cell resistance to oxidants; it exhibits multiple cell‐protective effects against a range of toxicities and chronic diseases associated with oxidative stress. (Ma, 2013). When cells are exposed to oxidative stress, GCL is rapidly activated by NRF2, contributing to NRF2‐mediated cell protection following oxidative stress (Krejsa et al., 2010). The NRF2 mRNA levels were increased under the condition of EXO^hCdHs^, and its target molecule, GCL, mRNA levels also increased (Figure 3c). Therefore, these pieces of data show that EXO^hCdHs^ treated cells can reduce oxidative stress, and the inhibition of ROS generation in EXO^hCdHs^ treated group is regulated by their antioxidant systems.

### 3.3 EXO^hCdHs^ protects against oxidative stress induced cell death of hepatocytes via MCL‐1 and BCL‐X_L_ signaling pathways

**Figure 4:**
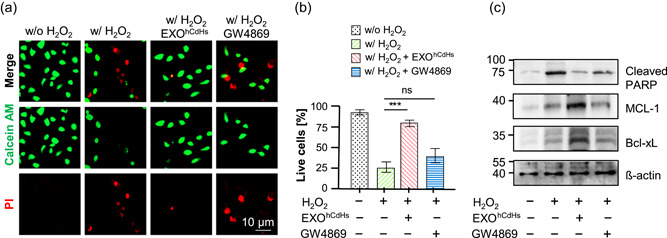
EXO^hCdHs^ inhibited oxidative stress induced cell death signaling of hepatocyte. Hepatocytes were treated with H_2_O_2_ for 6 hr in the presence or absence of EXO^hCdHs^. Cell viability was determined by calcein‐AM (green) and propidium iodide (PI; red) double staining. (a) Representative confocal images of live and dead cells. (b) Percentages of live cells. The values are means ± *SEM* from three independent experiments. ***p < .001 (w/H_2_O_2_ vs. w/H_2_O_2_ + EXO^hCdHs^), ns, nonsignificant (w/H_2_O_2_ vs. w/H_2_O_2_ + GW4869). Scale bar = 10 μm; unpaired, two‐tailed *t* test with Welch's correction. (c) The cleaved PARP protein and antiapoptotic protein levels such as MCL‐1 and Bcl‐xL were determined by western blot analysis [Color figure can be viewed at wileyonlinelibrary.com]

**Figure 5:**
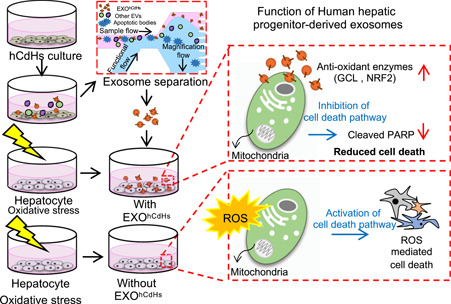
The antioxidant effect of EXO^hCdHs^ in H_2_O_2_‐exposed hepatocyte. Schematic summary underlying the apoptotic death of hepatocytes induced by H_2_O_2_ in the presence or absence of EXO^hCdHs^. EXO^hCdHs^ accelerates to secrete antioxidant enzymes such as GCL and NRF2 for inhibiting cell death when hepatocytes were damaged from oxidative stress [Color figure can be viewed at wileyonlinelibrary.com]

To better understand the function of EXO^hCdHs^, we analyzed the ROS induced cell death of hepatocytes treated with H_2_O_2_ (400 μM). Immortalized hepatocytes were pretreated with EXO^hCdHs^ (1:2,000, dilution in cell culture media) for 24 hr and were kept until 30–36 hr in the presence of H_2_O_2_. After 48 hr, a live and dead assay was conducted to assess the survival rates of cells treated with H_2_O_2_. As shown in Figure 4a, cell death of hepatocytes by oxidative stress was decreased by treatment with EXO^hCdHs^. Furthermore, the percentages of live cell were blocked by treatment with exosome‐depleted solution that was pretreated with GW4869, and there is no significant difference in the presence or absence of GW4869 (Figure 4b). These results were confirmed by detecting the amounts of cleaved PARP in hepatocytes by western blot analysis (Figure 4c). Cleaved PARP were decreased through treating with EXO^hCdHs^ compared to the nontreated group (Figure 4c). According to previous reports, the p53 and bcl‐2 family proteins, bax, and bcl‐2, as well as PUMA acted as downstream signaling effectors of ROS‐dependent inferences (J. Yang et al., 2017). Therefore, we checked the antiapoptotic protein expression, such as MCL‐1 and Bcl‐X_L_ protein. Protein expression levels are increased by treatment with EXO^hCdHs^ (Figure 4c). These findings are supported by the expression of MCL‐1 and Bcl‐XL regulating ROS induced cell death by treatment with EXO^hCdHs^. Finally, the schematic diagram showed that the ROS induced cell death is attenuated by EXO^hCdHs^ in hepatocytes (Figure 5).

## 4 DISCUSSION

In this study, we confirmed that EXO^hCdHs^ play an essential role in preventing oxidative induced cell death of hepatocyte. The survival of damaged hepatocytes was enhanced by treatment with EXO^hCdHs^, which promote the increase of the antioxidant gene expressions and decrease ROS production. Furthermore, these potential effects were decreased by adding the exosome that was pretreated with GW4869, followed by the acceleration of ROS production, thereby causing the decrease of cell viability. These findings show that exosomes may be related to the recovery process of ROS‐dependent antioxidant activity.

Oxidative stress has been considered as a conjoint pathological mechanism, and it contributes to initiation and progression of liver injury, which has been demonstrated in animal and human studies (Zhu, Wang, Zhang, & Guo, 2012). Risk factors include abuse of alcohol, drugs, viral infections and environment, which may induce oxidative stress in the liver. In turn this results in severe liver disease, such as alcoholic liver disease, nonalcoholic steatohepatitis and hepatocellular carcinoma (HCC; Cichoz‐Lach & Michalak, 2014) Recently, we reported that hCdHs were reprogrammed to bi‐potential progenitor cells from the hepatocytes by using small molecules, A83‐01 and CHIR99021 including HGF (Kim et al., 2019). Thus, our recent data show that EXO^hCdHs^ inhibited oxidative induced cell death in hepatocytes. Consistently, EXO^hCdHs^ activate NRF2 expression and induces downstream regulators such as GCL. The NRF2 is an emerging regulator of cellular resistance to oxidants and induces the expression of antioxidant response element‐dependent genes to regulate outcomes of oxidant exposure (Ma, 2013) and protects against oxidative stress in impaired primary biliary cholangitis (Wasik, Milkiewicz, Kempinska‐Podhorodecka, & Milkiewicz, 2017). Moreover, glutathione biosynthesis can be regulated by GCL, which has a catalytic subunit GCLC and is regulated by NRF2 (Zhong, Mishra, & Kowluru, 2013). The modulation of oxidative stress by reinforcing the antioxidant defense system, would be an effective therapeutic target to improve the performance of liver disease and liver transplantation.

Exosome‐mediated signaling has been found to be included in the regulation of cell protection and regeneration under various physiological and pathological conditions (Royo & Falcon‐Perez, 2012; Yanez‐Mo et al., 2015). The hepatocyte‐derived exosome can induce hepatocyte proliferation both in vitro and in vivo of the liver injury model by transferring sphingosine kinase 2 (SK2), causing enhanced synthesis of sphingosine‐1‐phosphate (S1P) to recipient cells (Nojima et al., 2016; Wu et al., 2018). Importantly, the large contribution of stem cell‐derived exosomes to cell protection against liver disease has been confirmed through inhibition of exosome cargo, suggesting that exosomes participate as chemotherapeutic agents. Similarly, exosomes derived from cancer cells not only regulate the proliferation and cell growth of tumor cells but also inhibit cell apoptosis by caspase‐3‐dependent cleavage of Bcl‐xL within the exosome (Vardaki et al., 2016). In particular, caspase‐3 signaling seems to be required for exosome uptake by target cells, activating the cell‐protective function (Vardaki et al., 2016; L. Yang, Wu, Wang, Luo, & Chen, 2013); however, the significance of this signaling pathway in the context of exosomes is not completely known yet. Consistent with these findings, our results support the hypothesis that CdHs mediates inhibition of oxidative induced cell death of hepatocytes, and perhaps also hepatocyte metabolism and other functions, suggesting that exosomes in the liver system could have significant clinical applications as biomarkers or therapeutic agents.

## 5 CONCLUSION

In the present study, EXO^hCdHs^ significantly improved hepatocyte cell viability; this effect was confirmed by depleting the exosome supply. The antioxidant effect of exosomes on damaged hepatocytes was related to inhibition of the oxidative stress induced cell death signaling pathway. Furthermore, exosomes could be functional to attenuate the cellular toxicity of hepatocytes as well as liver injury through decreased oxidative stress induced cell death signaling. These exosome‐mediated effects provide new insights into liver function in the liver system and indicate the potential of exosomes as a therapeutic agent.

## CONFLICT OF INTERESTS

The authors declare that there are no conflict of interests.

## AUTHOR CONTRIBUTIONS

S. H. performed in vitro culture, western blot analysis, ROS assay, and cytotoxicity measurement and drafted the manuscript. J. M. J. and J. H. Y. performed the establishment of hCdHs and J. M. J. immortalized hepatocytes, qRT‐PCR, and wrote the relevant sections in the manuscript. K. S. performed exosome separation analysis and NTA measurement. C. J. Y. performed transmission electron analysis and wrote the relevant sections under the supervision of H.S.J. S.H. wrote the manuscript with all authors' input. K. G. H., D. C., and J. W. H. designed the research and supervised the study. All authors read and approved the final manuscript.

## Supporting information

Supporting information
