# Supplementary material for: Exosomes derived from chemically induced human hepatic progenitors inhibit oxidative stress induced cell death
Source: Biotechnol Bioeng. 2020 Jun 30;117(9):2658–67. doi: 10.1002/bit.27447 (PMC7496643; doi:10.1002/bit.27447)
Supplement: Supplementary file 1 — Supporting information [file BIT-117-2658-s001.pdf]

Exosomes derived from chemically induced human hepatic progenitors inhibit oxidative stress induced cell death

Sujin Hyung[1†], Jaemin Jeong[2,3†], Kyusoon Shin[1,4†], Ju Young Kim[1,4], Ji-Hye Yim[2,3], Chan Jong Yu[5], Hyun Suk Jung[5], Kyung-Gyun Hwang[6], Dongho Choi[2,3]*, and Jong Wook Hong[1,4,7,8]*

[1]Center for Exosome & Bioparticulate Research, Hanyang University, Gyeonggi-do,15588, Korea
[2]HY Indang Center of Regenerative Medicine and Stem Cell Research, Hanyang University, Seoul 04763, Korea
[3]Department of Surgery, Hanyang University College of Medicine, Seoul 04763, Korea
[4]Department of Bionanotechnology, Graduate School, Hanyang University, Seoul, 04763, Korea
[5]Division of Chemistry and Biochemistry, Kangwon National University, Chuncheon, 24341, Korea
[6]Department of Dentistry/Oral & Maxillofacial Surgery, Collage of Medicine, Hanyang University, Seoul, 04763, Korea
[7]Department of Bionanoengineering, Hanyang University, Gyeonggi-do, 15588, Korea
[8]Department of Medical & Digital Engineering, Hanyang University, Seoul, 04763, Korea

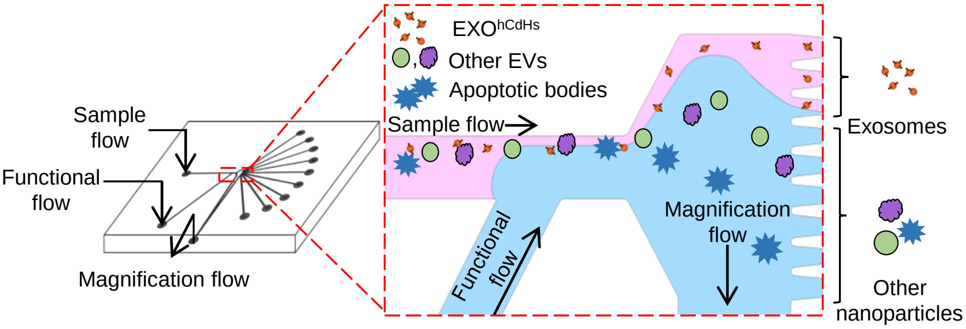

Supplementary Figure 1. Schematics of exosomes separated from hCdHs using the 'H' method. The conditioned medium of hCdHs applied to the 'H' method in which particles such as exosomes, microvesicles, and apoptotic bodies were isolated by dependent with particle size. The exosomes were automatically isolated and collected in the first three channels of nine channels through the 'H' method.

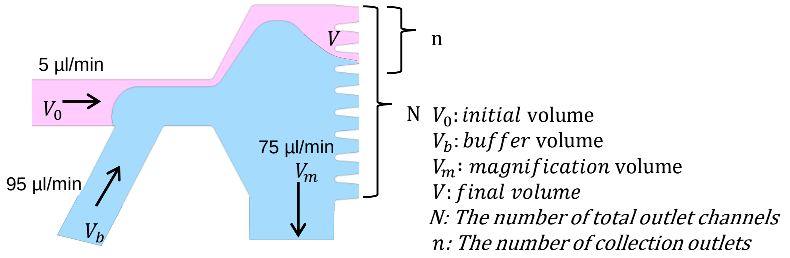

Supplementary Figure 2. Flow rates of sample, buffer, and magnification channels for exosome isolation. To isolate exosomes, the flow rate of injected sample and buffer solution were 5 µl/min and 95 ul/min, respectively, and the buffer removed by magnification was 75 µl/min.

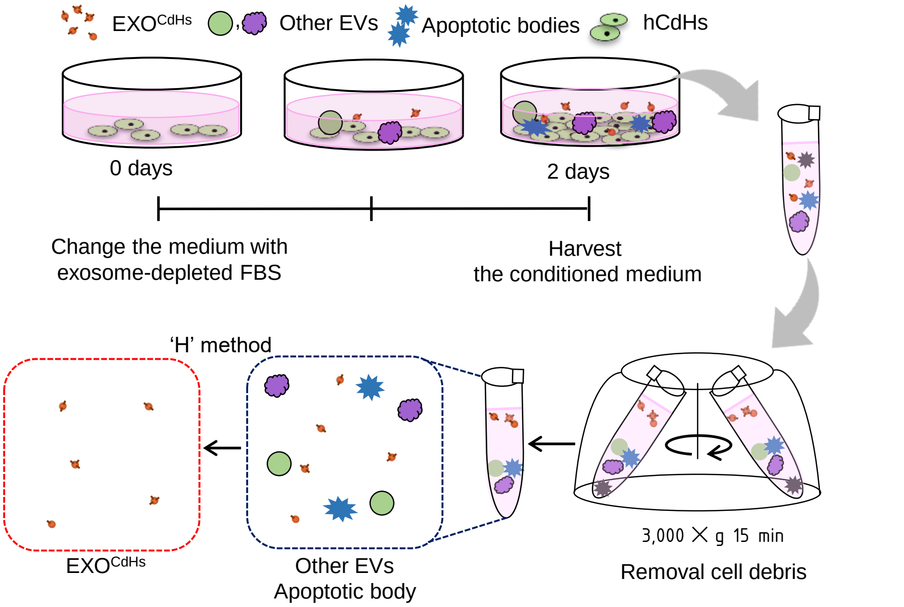

Supplementary Figure 3. Schematics of hCdHs cell culture procedure for exosomes separation. Cultured hCdHs were replaced with fresh cell culture medium containing exosome-depleted FBS were then incubated for 2 days. The conditioned medium of hCdHs was collected and centrifuged at 3000 x g for 15 min to remove cell debris.
